# Prenatal and Postnatal Manifestations of Congenital Chloride Diarrhea Due to a Heterozygote Variant of the *SLC26A3* Gene: A Case Report

**DOI:** 10.3389/fped.2021.758006

**Published:** 2021-12-20

**Authors:** Izabela Cendal, Agnieszka Szafrańska, Tomasz Fuchs, Dariusz Patkowski, Robert Smigiel, Barbara Królak-Olejnik

**Affiliations:** ^1^Department and Clinic of Neonatology, Wroclaw Medical University, Wroclaw, Poland; ^2^II Department of Gynecology and Obstetrics, Wroclaw Medical University, Wroclaw, Poland; ^3^Department of Pediatric Surgery and Urology, Wroclaw Medical University, Wroclaw, Poland; ^4^Department of Pediatrics, Division of Pediatrics and Rare Disorders, Wroclaw Medical University, Wroclaw, Poland

**Keywords:** congenital chloride diarrhea (CCD), infant diarrhea, chronic diarrhea, *SLC26A3* gene, case report

## Abstract

Congenital chloride diarrhea (CCD) is caused by a recessive mutation in the *SLC26A3* gene and characterized mainly by watery diarrhea, hypochloremia and metabolic alkalosis. Various different mutations in *SLC26A3* are responsible for the disease. In the prenatal period, the symptoms of CCD may include polyhydramnios, preterm labor and abdominal distension. The main feature of CCD is chloride-rich diarrhea, which leads to excessive loss of fluid and salt immediately after birth and is followed by weight loss and dehydration. Hyponatremia and hypochloremia are soon accompanied by hypokalemia and metabolic alkalosis. Untreated CCD is fatal even in the first weeks of life. Diagnosis is made by high fecal chloride concentrations in patients with serum electrolytes corrected by salt substitution and confirmed using genetic testing of peripheral blood samples. Here, we detail prenatal and postnatal manifestations of a preterm infant, born via Caesarian section, who was suspected to suffer intrauterine bowel obstruction. Upper median laparotomy was performed and no intestinal abnormalities found. The course of the neonatal period was complicated by severe diarrhea with hypochloremia, hyponatremia and metabolic alkalosis. Based on the patient's clinical picture and stool examination, a diagnosis of CCD was established. Mutation of the *SLC26A3* gene was confirmed using genetic testing.

## Introduction

Congenital chloride diarrhea (CCD), also known as DIAR1, is a rare autosomal recessive inherited disease. In available literature, cases described involved various mutations in the 26-member solute carrier family of proteins. To date, over 40 different mutations relevant to CCD lack robust evidence of phenotype–genotype correlation (1–3). The DIAR1 variant of CCD is caused by a recessive mutation in the A3 (*SLC26A3*), located on chromosome 7q22.3-31.1 gene and is characterized mainly by watery diarrhea, hypochloremia and metabolic alkalosis (OMIM 214700, ORPHA 53689). Other forms of congenital diarrhea include DIAR2 (OMIM 251850), caused by a mutation in the *MYO5B* gene (OMIM606540); DIAR3 (OMIM270420), caused by a mutation in the *SPINT2* gene (OMIM605124); DIAR4 (OMIM610370), caused by a mutation in the *NEUROG3* gene (OMIM604882); DIAR5 (OMIM613217), caused by a mutation in the *EPCAM* gene (OMIM185535); DIAR6 (OMIM614616), caused by a mutation in the *GUCY2C* gene (OMIM601330); DIAR7 (OMIM615863), caused by a mutation in the *DGAT1* gene (OMIM604900); DIAR8 (OMIM616868), caused by a mutation in the *SLC9A3* gene (182307); DIAR9 (OMIM618168), caused by a mutation in the *WNT2B* gene (OMIM601968); DIAR10 (OMIM618183), caused by a mutation in the *PLVAP* gene (OMIM607647) and DIAR11 (OMIM618662), caused by deletion of the intestine critical region (ICR) on chromosome 16p13, thus resulting in loss of *PERCC1* gene (OMIM618656) function (4). The mutations impair the intestinal active transport functionality of Cl^−^/HCO3^−^ as it is mainly expressed in the apical brush border of the colonic and ileal epithelium (5, 6).

Chiefly manifesting in the first days of life as a watery diarrhea with high chloride levels (>90 mmol/l) and a low pH, CCD rapidly leads to hypochloremia, hypokalemia, metabolic alkalosis and dehydration. Other laboratory abnormalities in the neonatal period may include hyponatremia, hyperphosphatemia, hypermagnesemia, hyperuricemia, hypercreatinemia, hyperreninemia and hyperaldosteronism. Some reports highlight delayed meconium excretion and jaundice (3, 7). As early as the prenatal period, importantly, signs similar to those seen in the setting of intestinal obstruction, such as dilated intestinal loops on ultrasound, may be apparent. Polyhydramnios, manifesting due to intrauterine watery diarrhea, as well as pre-term birth, may also be common (3, 6–10). Watery stools in CCD are frequently confused with urine, often contributing to a delay diagnosis (2). Conditions such as microvilli inclusion disease, cystic fibrosis, Bartter syndrome and congenital tufting enteropathy may present clinically with symptoms similar to those of CCD (9, 11). Undiagnosed CCD is a serious illness and there are only rare cases of undiagnosed children surviving past the first year of life (9).

Complications of CCD include kidney damage and associated electrolyte abnormalities, enteritis, impaired sweat secretion, hyperuricemia and decreased male fertility (7, 10). In addition, it is currently understood that worsening renal function and elevated plasma renin levels predispose CCD patients to hypertension (12).

## Case Report

**Figure 1:**
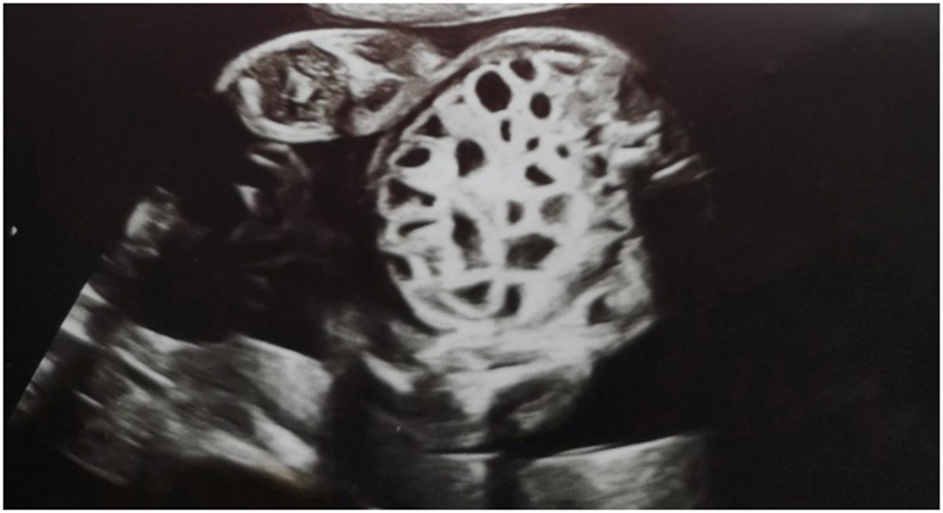
Ultrasound views at 24 weeks and 4 days of gestation showing multiple dilatation of the bowel loops filled with fluid.

A second-pregnancy male neonate (the previous pregnancy ended in miscarriage) was delivered by emergency cesarean section at 32 and 4/7 weeks gestational age (GA) due to suspected fetal intrauterine bowel obstruction. Birth weight of the baby was 2260 g (50–90 percentile). In the 28th week of pregnancy, hospitalization of the mother was required due to detected polyhydramnios and an increased risk of premature birth; 1000 cm^3^ of amniotic fluid was decompressed and prenatal steroid therapy was initiated. The course of pregnancy was further complicated by anemia (hemoglobin 6.7 g/dl) that was treated with oral iron and an upper respiratory tract infection at 30 weeks GA that was treated symptomatically. Prenatal ultrasound at 24 and 4/7 weeks GA revealed dilatation of multiple fluid-filled bowel loops (i.e., the honeycomb sign; Figure 1).

The preterm infant was nevertheless clinically stable and required only support in the delivery room; physical examination revealed abdominal distention and no rectal meconium was noted on catheterization. A rectal enema, however, yielded watery content. The infant's weight loss of 340 g (15% of birth weight) on the first day of life despite age-appropriate fluid therapy was perplexing. Normal peristalsis was noted.

**Figure 2:**
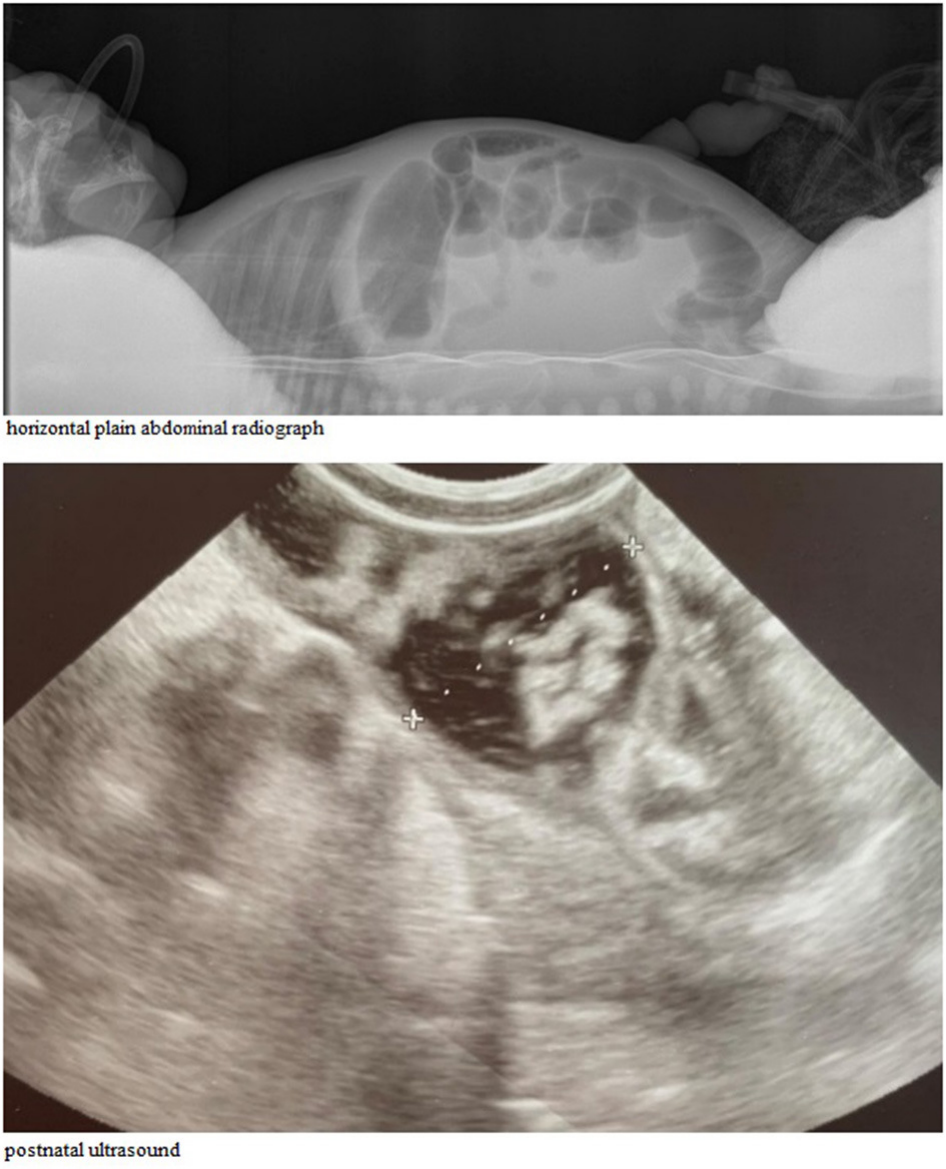
Abdomen X-ray in the first day of life, horizontal radius **(Top)**. Abdominal ultrasound examination showing significantly distended intestinal loops of the newborn **(Lower)**.

**Figure 3:**
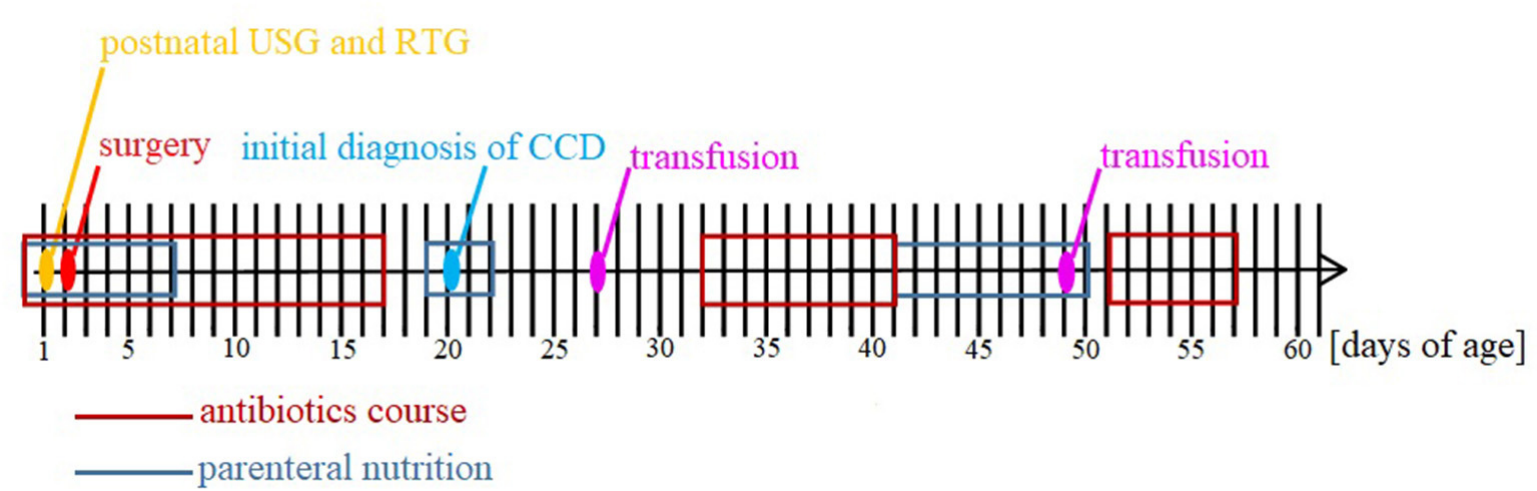
Timeline of hospitalization.

**Figure 4:**
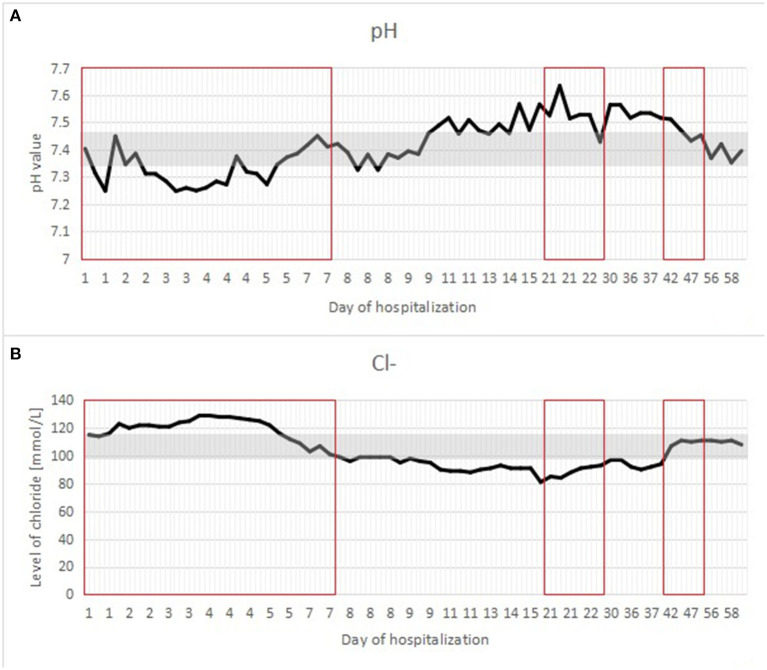
**(A,B)** A graph showing changes in the patient's blood pH and in the level of chloride during hospitalization. The reference range is presented in a gray frame. The red box marks the periods of parenteral nutrition.

A postnatal ultrasound and horizontal plain abdominal radiograph (Figures 2, 3) confirmed the presence of fluid levels consistent with lower gastrointestinal obstruction. There was no passage of stool and surgery was undertaken on the second day of life. During the procedure, no abdominal pathologies were noted. The bowels were equally distended and filled with air and clear liquid. No meconium was visible through the bowel wall, which was worrisome, especially in conjunction with no stool passage after birth. Post-operative, the infant was pulmonary and circulatory unstable and required intensive care including mechanical ventilation. In this period he was mainly fed parentally, and no electrolyte disturbances were noted. As the infant stabilized, feeds were reintroduced and from 7th day they were primarily oral, whereafter pronounced hypochloremic alkalosis evolved (see Figure 4- A graph showing changes in the patient's blood pH and in the level of chloride during hospitalization. The reference range is presented in a gray frame. The red box marks the periods of parenteral nutrition). Cystic fibrosis has been ruled out by genetic testing. Diarrhea was not present, on the contrary constipation was suspected but upon rectal catheterisation an excessive amount of stool was noted. CCD was first considered at day 20 and supplementation with NaCl and KCl initiated, leading to partial normalization of electrolytes.

After initial improvement, the patient's condition deteriorated once again and he suffered abdominal bloating, weight loss and irritability; positive fecal occult blood was also noted. The diagnosis of sepsis was made on the basis of blood cultures. Rehydration, total parenteral nutrition, omeprazol and antibiotics consistent with sensitivities were administered. After markers of infection decreased, oral re-feeding was initiated, with the mother's milk. Breast milk intolerance, however, was noted, despite the mother's adherence to a non-dairy diet. Introduction of a hypoallergenic amino acid formula allowed for the cessation of parenteral nutrition at 50 days of age. The patient furthermore required an oral NaCl/KCl salt-substitution therapy.

Prenatal intestinal distention erroneously was an indication for laparotomy, but the patient developed abdominal distension, diarrhea, hypochloremic alkalosis, hyponatremia, and intermittent hypokalemia in the following days of life. Despite prematurity and full-blown sepsis, which hindered unequivocal diagnosis, the performed imaging and laboratory examinations led to suspicion of CCD. The diagnosis was definitively confirmed by genetic testing.

After 61 days of hospitalization, the patient was discharged in good condition and recommended to be followed-up at the neonatal outpatient clinic and gastroenterological department. Feeding with hypoallergenic amino acid formula (Neocate LC) supplemented with 0.9% NaCl (18 ml per portion) was prescribed.

As of the time of this report, the 6-month-old infant was being managed in the neonatal outpatient clinic. The baby was properly growing and gaining weight, corrected for 4 months of age. On recent review, no abdominal distension, pathological abnormalities of stool or irritability were noted. Up to 12 soft stools per day were most recently reported, with the mother describing the baby's bowel movements as profuse about twice per day. The longest interval between defecation was 2–3 h. Most recently, the greatest problem was reported to be diaper dermatitis despite proper care and frequent diaper changes. For this reason, an attempt was made to use *ad hoc* oral cholestyramine to reduce diarrhea. Studies have reported the effectiveness of cholestyramine in patients with CCD, possibly due to its ability to bind bile acids that reach the colon with increased ileal effluent and stimulation of an additional Cl^−^ secretory response (13). Hyperhidrosis was also diagnosed in the child. Control laboratory examinations revealed electrolyte levels to be within the normal range while abdominal ultrasound revealed distension of the large intestine. No signs of nephrolithiasis were noted and the infant's diet was expanded in accordance with his corrected age. Neurological examination revealed initially overstretched positioning and motor pattern, behavioral disorders, increased irritability and tearfulness. Torticollis was noted on physical examination while MRI revealed widening of the cerebral space, which does not increase in control ultrasound. Despite a paucity of patients with CCD in Poland, the parents of this child managed to establish contact with two other families caring for children suffering CCD and consider this a very helpful measure of support in care of the infant.

To avoid infections that can cause life-threatening dehydration, patients with CCD should always be up to date with their vaccinations. Auxological parameters and neuropsychological development should be regularly assessed–in the study by Di Meglio et al. (13), a high percentage of neuropsychomotor disturbances was found in CCD patients, without any significant genotype/phenotype relationship. Poor treatment compliance and delayed diagnosis were the two most important negative prognostic factors; high rates of failure to thrive, psychomotor delay, and renal impairment have been apprised in these patients. In adult patients, an important aspect of the clinical management of CCD is adequate replacement therapy, and the monitoring and treatment of intestinal and parenteral complications, such as chronic kidney disease, hyperuricemia, spermatoceles, and inflammatory bowel disease. For this reason, patients with CCD should be constantly monitored by a gastroenterologist and a nephrologist (13).

## Genetic Testing

This study was conducted utilizing next-generation sequencing technology. Sequences of enriched DNA regions were read using a HiSeq 4000 sequencer (Illumina) at a reading length of 2 x 150 nucleotides. Genetic variants were identified using the Burrows–Wheeler Aligner software package. This study analyzed exon coding sequences along with 10–20 nucleotide intron flanks of the following genes: *DGAT1, EPCAM, GUCY2C, LCT, MYO5B, NEUROG3, SLC26A3, SPINT2, TMPRSS15* and *TTC37*.

Genetic analysis revealed pathology in both alleles of the *SLC26A3* gene in the variant ENST00000340010: c.2024_2026dupTCA resulting in amino acid position alteration in the p.Ile675dup protein. The *SLC26A3* gene is associated with CCD and is inherited in an autosomal recessive manner; both copies of the gene must thus be damaged in order for symptoms to manifest. The patient was found to be homozygous, meaning that both copies of the gene were damaged. Familial genetic testing revealed carriage of a variant in one allele in both parents.

## Discussion

We report a rare case of CCD in preterm male neonate. Our patient had abdominal distension, diarrhea and hypochloremic alkalosis. Other clinical features included hyponatremia, and intermittent hypokalemia. Because of the rarity of CCD cystic fibrosis was suspected on admission, and the diagnosis of CCD was not initially considered. However, after he had improved after NaCl and KCl supplementation and after excluding cystic fibrosis by genetic testing, CCD suspicion was raised. The diagnosis was confirmed by genetic testing. Since our case study focuses on a single patient, more studies are needed to make clear recommendations for dealing with suspected CCD. A high index of suspicion is needed to both make the diagnosis, and avoid unnecessary medical treatments associated with incorrect diagnoses. This case will help raise awareness of CCD to improve patient outcomes.

CCD manifests prenatally as polyhydramnios, fetal intestinal distension and frequently preterm delivery. CCD is most common among inhabitants of Kuwait and Saudi Arabia (1:3200–1:5000) and slightly less common in Finland (1:30 000–1:40 000). In Poland, CCD has an estimated prevalence of 1:200 000 (8, 14).

The most important clinical sign of CCD is the presence of chronic diarrhea. However, as this study and other examples in literature report, its presence, especially in the neonatal period, is not always noted and is often masked by other disorders (e.g., infections, prematurity). In addition, it is exclusion of typical causes of diarrhea that leads to the recognition of the functional character of CCD. Although elevated fecal chloride ion concentration (>90 mmol/l) is pathognomonic for CCD, this test is not routinely performed and often further delays diagnosis of CCD. Urinary chloride concentration thus warrants measurement, and is typically noted to be low (<110 mmol/l) in the setting of hypochloremia associated with CCD. Although CCD can typically be diagnosed clinically as in this presented case, utilization of molecular testing allows for definitive diagnosis of the condition and provides data valuable for genetic counseling.

Both pre- and postnatal symptoms suggest gastrointestinal obstruction, a major reason for performing exploratory laparoscopy or invasive imaging procedures. In this case, both pre- and postnatal signs interestingly suggested severe lower intestinal obstruction similar to that frequently observed in conditions such as Hirschsprung disease. The polyhydramnios noted before delivery, gastrointestinal discharge, distended abdomen, and lack of stool passage were reasons for performing diagnostic procedures and eventually surgery. Diarrhea may well be expected to have occurred early in the course of the illness, although it presented later. Thus, ambiguous symptoms in early life present a significant diagnostic dilemma.

If CCD is suspected based on prenatal imaging, MRI is warranted. Several prenatal ultrasound findings help to distinguish CCD from lower intestinal atresia: generalized bowel dilatation from the rectum to the small intestine and normal peristalsis in the distended bowel. Moreover, T1-weighted MRI typically reveals hypointense fluid accumulation within the bowel (meconium is normally hyperintense). Prenatal diagnosis is possible and allows for avoidance of surgery in the newborn as well as appropriate management soon after birth (6).

To avoid dehydration and electrolyte imbalances, replacing salt with NaCl and KCl solutions as needed produces favorable long-term results in CCD patients. In infancy, solutions of 0.7% NaCl and 0.3% KCl should be used, while after the first 3 years of life, more concentrated solutions of 1.8% NaCl and 1.9% KCl should be used. The optimal dose of chloride ions should range from 6 to 8 mmol/kg/day for infants and 3 to 4 mmol/kg/day for elderly patients (15). In previously described cases, patients were still found to produce 7–8 watery stools per day after many years of follow up, although their development was found to be normal and no growth deficits were noted. Treatment with NaCl and KCl solutions, however, is life-long (10, 11). Captopril has been considered to be a potential therapeutic alternative. A study by Bin Islam et al. (16) reported that initiation of captopril resulted in a decrease in the amount of watery content of diarrheal stool as well as an overall decrease and change in the amount of stool excreted from an average of 500 ml of watery stool per day to 50 ml of semi-solid stool per day. KCl and NaCl supplementation was not required when captopril was administered (16).

CCD typically manifests with intestinal obstruction and necrotizing enterocolitis. Moreover, overlapping infections and respiratory distress syndrome of the newborn make the diagnosis of primary CCD more difficult. Determination of ion levels and acid-base parameters in the first week of life as well as in the postoperative period typically reveal metabolic acidosis, hypochloremia (up to 129 mmol/l) and hypernatremia (up to 150 mmol/l). Hypochloremic alkalosis was first reported in a patient closely monitored with daily electrolyte determinations at 9 days of age, after 2 days of enteral nutrition. In cases of patients suffering diarrhea complicated by hypochloremic alkalosis, stool examination should be performed as soon as possible to obtain levels of excreted electrolytes. Prior to definitive diagnosis of CCD in newborns, appropriate supplementation, which may prevent developmental problems and allow for proper neonatal weight gain, should be started.

Our patient was diagnosed with pathology in the *SLC26A3* gene (c.2024_2026dupTCA), which, as reported in previous studies, is characteristic in half of Polish CCD patients (8). Of note, however, only a few cases of this disease have been described in Poland, as it occurs rarely and thus always presents a diagnostic dilemma. Diagnosis in the neonatal period allowed the patient to be discharged in good condition with oral supplementation, and currently good weight gain. However, the patient's future is uncertain and there is a risk of repercussions in the future. Recent observations of the child during visits to the neonatal outpatient clinic confirm proper development and a reduction in the number of watery stools. Positive clinical results likewise suggest properly administered NaCl supplementation. Although CCD is an exceedingly rare illness, clinicians should nevertheless be cautious and consider it as a possible diagnosis when patients present with chronic diarrhea and hypochloremic alkalosis.

## Data Availability Statement

The datasets presented in this article are not readily available due to ethical and privacy restrictions. Requests to access the datasets should be directed to the corresponding author.

## Ethics Statement

Written informed consent was obtained from the minor(s)' legal guardian/next of kin for the publication of any potentially identifiable images or data included in this article.

## Author Contributions

AS organized the database. IC, AS, TF, DP, RS, and BK-O wrote sections of the manuscript. All authors contributed to manuscript revision, read, and approved the submitted version.

## Conflict of Interest

The authors declare that the research was conducted in the absence of any commercial or financial relationships that could be construed as a potential conflict of interest.

## Publisher's Note

All claims expressed in this article are solely those of the authors and do not necessarily represent those of their affiliated organizations, or those of the publisher, the editors and the reviewers. Any product that may be evaluated in this article, or claim that may be made by its manufacturer, is not guaranteed or endorsed by the publisher.
